# Corrective adjustment methods for relative age effects on French young swimmers’ performances

**DOI:** 10.1371/journal.pone.0283229

**Published:** 2023-04-24

**Authors:** Audrey Difernand, Quentin De Larochelambert, Robin Pla, Kilian Barlier, Andy Marc, Samuel Ferri, Olivier Dupas, Juliana Antero, Jean-François Toussaint, Adrien Sedeaud

**Affiliations:** 1 Institut de Recherche bio-Médicale et d’Épidémiologie du Sport (IRMES), Institut National du Sport, de l’Expertise et de la Performance (INSEP), Paris, France; 2 URP 7329, Université Paris Cité, Paris, France; 3 French Swimming Federation, 92110, Clichy, France; 4 Centre d’Investigation en Médecine du Sport, Assistance Publique—Hôpitaux de Paris, Hôtel-Dieu, Paris, France; Universidade Federal de Minas Gerais, BRAZIL

## Abstract

**Background:**

This study aimed to identify a Relative Age Effect (RAE) among French young swimmers and apply corrective adjustment procedures to rebalance performances according to categories and events.

**Methods:**

5,339,351 performances of French swimmers aged 10 to 18 were collected between 2000 and 2019. Birth quarters distribution was examined according to competitiveness level (‘All’, ‘Top50%’, ‘Top25%’ and ‘Top10%’), event and age category. A linear relationship between the distribution of performances and calendar days provides a calibration coefficient allowing to rebalance performances by considering the effect of RAE for each event. Then, adjusted performances are recalculated using this coefficient, the initial performance and the relative age.

**Results:**

Proportion of swimmers born in the first quarter was higher than the proportion of those born in the last quarter for all events and strokes (p < 0.01). RAE increases with the competitiveness level for all events. Indeed, among ’All’ 12 years old 50m freestyle swimmers, the proportion born in the first quarter is 30.9% *vs* 19.2% in the fourth quarter, while among the “Top10%”, 47.5% were born in the first quarter *vs* 10.3% in the last one. (p-value < 0.01). In average, each day represents a gap of 0.008 second, resulting in a difference of almost 3 seconds over a year. This tool is validated by comparing swimmers who have performed at least twice in a season. It provides a day by day rebalancing method for all swimming events and age categories.

**Conclusions:**

Relative age effect is present among French young male and female swimmers, and is strengthened by competitiveness level. A new corrective adjustment procedure to rebalance performances considering categories and events is proposed and validated. By applying such a tool, we are able to reveal the full potential of swimmers and make it possible to compare them at the same relative age.

## Introduction

Selection processes in sports are organized by age categories [1]. However, athletes born right after the cutoff date are almost one (in case of annual selections) or two categories older (if selections occur every two years) than the ones born shortly before the cutoff date [2]. This variation inside a same age-category can lead to an asymmetric repartition of athletes with respect to birth months, with an overrepresentation of those born at the beginning of the competitive year. This phenomenon is called the Relative Age Effect (RAE) [2]. The RAE has been shown for both sexes [3] and in multiple sports like ice hockey, football, volley-ball, basket-ball, baseball, rugby, tennis, track-and-field [4] and swimming [5–8]. This phenomenon is commonly transient: exacerbated in the youngest categories, it wanes at older ages [7,9]. Indeed, a study conducted within FC Barcelona on popular team sports from U10 to senior category showed that the RAE is lower among seniors, in male’s basketball, handball, futsal and football as well as in seniors female’s football than in younger categories^9^. Another study among young top-level swimmers has shown that there was no RAE at 16 whereas, there was a medium effect size at 13 ^7^.The RAE also exhibits potentiation effects with level of competitiveness: the higher the level, the greater the effect [7].

The RAE generates biases. First, the difference in age, which may be as large as 365 days for a one-year category (or 730 days for a two-year category), generates differences in physical, anthropometric and life experience. These differences provide a significant advantage during selections [4,10,11]. Second, selection processes are oriented toward current performance level rather than the potential one, *ie*. the future performances [12]. Third, RAE also directly acts on athlete’s motivation. Lack of performance can lead to a decline in motivation among athletes and in some cases to dropout. [6,13]: among the underlying causes of dropout in sports, RAE and birth quarters play important roles [14–16].

Solutions have already been settled to compensate for RAE: Corrective adjustment procedures were constructed from the regressed relationship between chronological age and objective performance measures in swimming [7,10,17], track-and-field [18] and ski [19]. Romann and Cobley determined the regression coefficient between performance time and relative age in Australian 100m Freestyle [7]. These studies removed RAE by verifying that the proportions in the birth quarters were not significantly different, but they did not reveal the impact of the rebalancing methods on the individual readjusted performances. For swimmers and coaches, assessing rebalanced individual performance could reduce the inequalities caused by age differences, improve motivation and participation in swimming and also build on the potential that resides in each individual.

The aims of the present study were: 1) to measure the presence of RAE in all individual swimming events and levels of competitiveness; 2) to determine and apply a corrective adjustment method for all events and age categories; 3) to rebalance the individual potential performance of athletes considering the existing RAE weighted by age category and event; and 4) to reveal the potential readjusted performances.

## Materials and methods

### Studied population

The dataset is composed of French female and male swimmers aged 10 to 18 years old. All swimmers competed at least once between 2000 and 2019 at a local, departmental, regional, national or international level in 25 meters or 50 meters swimming pools. The included events were 50m, 100m, 200m, 400m, 800m, 1500m Freestyle, 50m, 100m, 200m Backstroke, Breaststroke, Butterfly, 200m and 400m Individual Medley. Birth date, competition date, performance in seconds, stroke, size of pool and sex data were collected for each swimmer.

Each swimmer was present in the dataset, with his/her best performance appearing once per age category. The exact age of each swimmer at the time of a competition was calculated as the difference between the competition date and the birthdate. The relative age gap was defined by the time separating the last birthday and the competition date. It could be seen here as a time indicator in months depending on the exact age when comparing multiple swimmers.

### Ethics statement

The dataset was provided by the French Swimming Federation. All data were reported anonymously. This study was designed and monitored by the IRMES (Institut de Recherche bio-Médicale et d’Epidémiologie du Sport) scientific committee. Data collection was compliant with the General Data Protection Regulations applied in the European Union. A declaration of the study was made and approved by the Commission Nationale de l’Informatique et des Libertés (CNIL) with the following registration number: 2223498 v0.

### Identifying the relative age effect

Swimmers’ quarters of birthdate (Q1: born in January-March, Q2: April-June, Q3: July-September, and Q4: October-December) were created. The Top10%, Top25%, Top50%, and the all swimmers’ (Top100%) performance groups were created. Chi-square tests of adequacy to a uniform law were used to assess the significance of the different distributions among birth quarters; odds ratios were computed to compare the proportion of swimmers born in the first and last quarters. Significance thresholds were set at α = {0.1, 0.05, 0.01} (respectively *, **, *** in the results section).

### Corrective adjustment method

Linear regressions at all ages, for all events were run between the relative age gap and the performance mean. This method by age category was set up to quantify the RAE by age category, event and sex. Relative age gap was considered as the explicative variable to remove RAE linked to the competition dates. The calculated slope regression coefficient was then used to rebalance performance times and compare all of them at the exact same age. To calculate this time, the slope coefficient was multiplied by the time separating the competition date and the next birthday. As competitions were held all year through, the relocated performance had to be independent from the birthdate and competition date. This calculated time was then removed from the original time to obtain the adjusted performance time. A second linear regression between the relative age gap and the mean of adjusted performances was computed to test if the regression coefficient become zero (if not, the readjustment treatment would not be effective).


$$T_{adj}=T_{0}-c*m$$

*T*_*adj*_: adjusted performance time

T_0_: initial performance time

c: slope coefficient

m: time until the next birthday in days

To test the relocation methods, swimmers with two performances in a same year of age were considered. The two performances were separated by at least 6 months because we considered this period as sufficient to generate a progression. Relocation methods were applied to the first performance while considering the age difference between first and second performance. The relocated first performance was therefore obtained from the first performance, the rebalancing coefficient and the time delta separating the two performances.


$$T_{r}=T_{1}-c*(T_{2}-T_{1})$$

*T*_*r*_: rebalanced performance time

T_1_: initial performance time

T_2_: second performance time

c: slope coefficient

The Kolmogorov-Smirnov test was applied to check for normal distribution. As the sample did not follow the normal distribution hypothesis, a non-parametric Friedman test was performed to detect a significant difference between the three types of performance. We assumed the null hypothesis that the three types of performance follow the same distribution. For each age-group, it was not the case meaning that there was one type of performance that was different from the other ones. Therefore, a Wilcoxon test with Bonferroni correction (with the alpha value adjusted) was performed for each pair of possible performances to reveal which performance type was significantly different from the others.

All statistical analyses were performed using Python (version 3.8.5; Python Software Foundation, Delaware, United States).

## Results

### Identifying the relative age effect

1 343 095 performances in 50m pools (48.93% male, 51.07% female) and 3 996 256 performances in 25m pools (48.13% male, 51.87% female) were analyzed. An overview of the birth quarters distribution for each male and female event in 50m and 25m pools respectively is presented on S1 **and S2 Tables** in S1 File. When comparing the proportions born in Q2, Q3 and Q4, the proportion born in Q1 is always higher than the others. For example, in male breaststroke (50m pools), 31.4%, 31.34% and 31.56% were born during Q1 for 50m, 100m, 200m events respectively, *vs* 18.73%, 18.73% and 18.92% during Q4 (p < 0.01). The distribution of birth quarters according to age category and levels of competitiveness are showed on **Table 1**. The proportion of athletes born in Q1 is significantly higher than in all other quarters. Except for 13 years old category among “All”, proportions decrease from the first quarter to the last one for each age category. As the level of competitiveness increases, the proportion within the first quarter of birth is greater, to the detriment of the fourth quarter, as indicated by ORs. Indeed, for ‘All’, the OR between Q1 and Q4 is maximal for the youngest category, with 1.99. However, for ‘top10%’, the OR oscillates between 3.59 (17 years old) and 7.85 (12 years old). This means that it is at least 3.59 times more likely to find a swimmer born in Q1 than in Q4 in the ‘top10%’ among the 17 years old swimmers.

**Table 1:** Birth quarters distribution according to age and level of competitiveness of 50m Freestyle male swimmers in 50m pool.

Age category	Top %	Total	Q1 (%)	Q2 (%)	Q3 (%)	Q4 (%)	χ^2^	p-value	OR	p	95% CI
10	100	4439	1400 (31.54)	1176 (26.49)	1029 (23.18)	834 (18.79)	154.26	***	1.99	**	[1.79; 2.22]
11	100	6201	1838 (29.64)	1627 (26.24)	1480 (23.87)	1256 (20.25)	116.24	***	1.66	**	[1.51; 1.82]
12	100	8720	2692 (30.87)	2296 (26.33)	2054 (23.56)	1678 (19.24)	249.30	***	1.87	**	[1.73; 2.02]
13	100	10723	3282 (30.61)	2625 (24.48)	2659 (24.8)	2157 (20.12)	238.51	***	1.75	**	[1.63; 1.88]
14	100	13358	4136 (30.96)	3542 (26.52)	3177 (23.78)	2503 (18.74)	419.69	***	1.95	**	[1.83; 2.08]
15	100	15460	4771 (30.86)	4274 (27.65)	3503 (22.66)	2912 (18.84)	524.55	***	1.92	**	[1.82; 2.03]
16	100	13385	3935 (29.4)	3796 (28.36)	3071 (22.94)	2583 (19.3)	360.77	***	1.74	**	[1.64; 1.84]
17	100	11657	3290 (28.22)	3299 (28.3)	2662 (22.84)	2406 (20.64)	209.72	***	1.51	**	[1.43; 1.6]
10	50	2219	791 (35.65)	590 (26.59)	479 (21.59)	359 (16.18)	182.27	***	2.87	**	[2.46; 3.34]
11	50	3100	1100 (35.48)	842 (27.16)	657 (21.19)	501 (16.16)	256.92	***	2.85	**	[2.51; 3.24]
12	50	4360	1636 (37.52)	1147 (26.31)	935 (21.44)	642 (14.72)	482.66	***	3.48	**	[3.11; 3.89]
13	50	5361	1968 (36.71)	1332 (24.85)	1243 (23.19)	818 (15.26)	504.64	***	3.22	**	[2.91; 3.57]
14	50	6679	2415 (36.16)	1846 (27.64)	1474 (22.07)	944 (14.13)	689.62	***	3.44	**	[3.14; 3.77]
15	50	7730	2759 (35.69)	2208 (28.56)	1642 (21.24)	1121 (14.5)	777.19	***	3.27	**	[3.01; 3.55]
16	50	6692	2234 (33.38)	1937 (28.95)	1457 (21.77)	1064 (15.9)	479.35	***	2.65	**	[2.43; 2.89]
17	50	5828	1860 (31.91)	1662 (28.52)	1253 (21.5)	1053 (18.07)	280.90	***	2.13	**	[1.95; 2.33]
10	25	1109	413 (37.24)	288 (25.97)	241 (21.73)	167 (15.06)	115.47	***	3.35	**	[2.69; 4.18]
11	25	1550	581 (37.48)	430 (27.74)	310 (20.0)	229 (14.77)	181.62	***	3.46	**	[2.89; 4.14]
12	25	2180	899 (41.24)	557 (25.55)	440 (20.18)	284 (13.03)	375.42	***	4.69	**	[4.01; 5.48]
13	25	2680	1127 (42.05)	632 (23.58)	595 (22.2)	326 (12.16)	498.89	***	5.24	**	[4.53; 6.06]
14	25	3339	1322 (39.59)	920 (27.55)	697 (20.87)	400 (11.98)	542.27	***	4.82	**	[4.22; 5.5]
15	25	3865	1503 (38.89)	1113 (28.8)	766 (19.82)	483 (12.5)	603.64	***	4.46	**	[3.96; 5.02]
16	25	3346	1180 (35.27)	977 (29.2)	697 (20.83)	492 (14.7)	329.79	***	3.16	**	[2.79; 3.58]
17	25	2914	993 (34.08)	832 (28.55)	615 (21.11)	474 (16.27)	217.33	***	2.66	**	[2.34; 3.03]
10	10	443	183 (41.31)	122 (27.54)	84 (18.96)	54 (12.19)	83.82	***	5.07	**	[3.58; 7.18]
11	10	620	244 (39.35)	184 (29.68)	118 (19.03)	74 (11.94)	107.69	***	4.79	**	[3.56; 6.44]
12	10	872	414 (47.48)	207 (23.74)	161 (18.46)	90 (10.32)	266.83	***	7.85	**	[6.13; 10.05]
13	10	1072	499 (46.55)	237 (22.11)	225 (20.99)	111 (10.35)	301.57	***	7.54	**	[6.0; 9.47]
14	10	1335	542 (40.6)	366 (27.42)	273 (20.45)	154 (11.54)	240.93	***	5.24	**	[4.25; 6.46]
15	10	1546	621 (40.17)	443 (28.65)	306 (19.79)	176 (11.38)	281.95	***	5.23	**	[4.31; 6.34]
16	10	1338	482 (36.02)	403 (30.12)	276 (20.63)	177 (13.23)	163.46	***	3.69	**	[3.02; 4.51]
17	10	1165	416 (35.71)	354 (30.39)	239 (20.52)	156 (13.39)	139.13	***	3.59	**	[2.9; 4.44]

(Top %: Top percentage, N: Number of observations, Q1-4: Birth quarters 1 to 4, χ^2^: Chi-Square value, ***: P < 0.01, **: P <0.05, OR: Odds ratios between Q1 and Q4, 95% CI: 95% Confidence Interval).

### Corrective adjustment method

**Fig 1:**
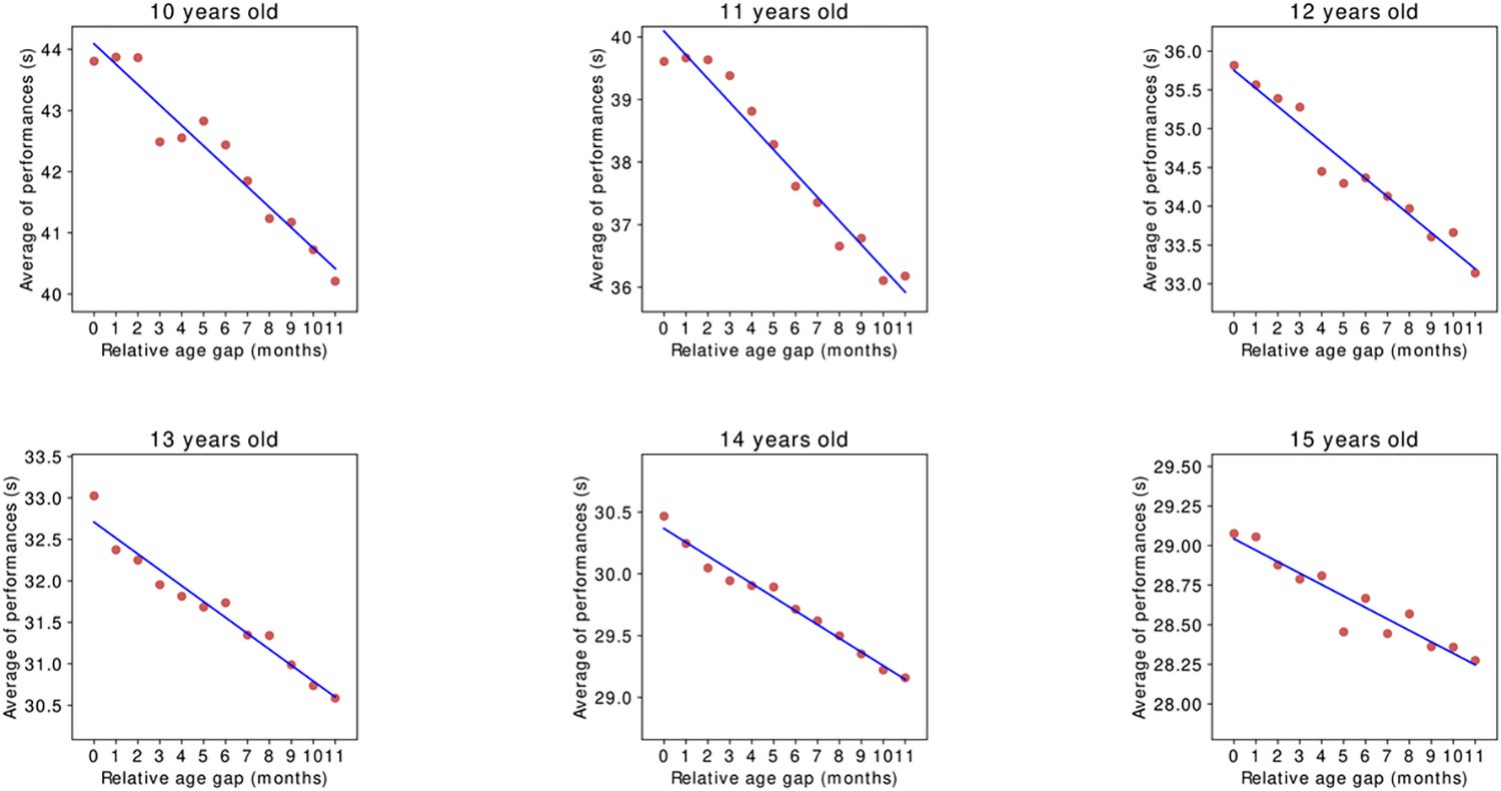
*Performance means and relative age gap by age on 50m Freestyle male swimmers in 50m pools for* 10-years-old *to* 15-years-old *categories*.

The linear relationship between the performance mean (in seconds) and the relative age gap at each age between 10 and 16 years old in 50m Freestyle male swimmers in 50m pools is displayed on **Fig 1**. The slope coefficient is negative at all ages: *ie*. in average, older swimmers are faster in all age categories. For example, in 10-years-old category, each day of relative age gap provides a performance reduction of 0.011 second. This can be translated into a 4 seconds gap for a 365 days difference in birthdates (being born on January 1^st^ *vs* December 31^st^). It is worthwhile to underline an order of magnitude in the decreasing evolution of the slope coefficient: from -0.013 seconds per day in 11 years old group to -0.002 per day in 15 years old group.

**Fig 2:**
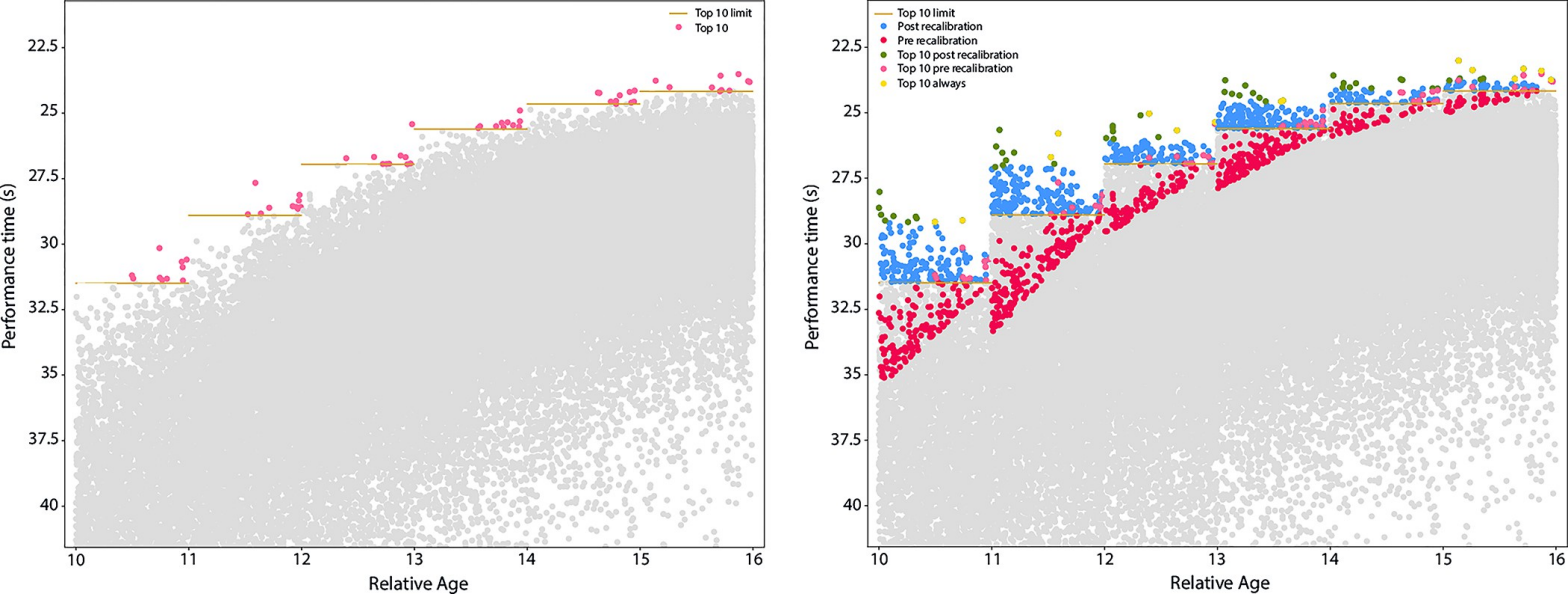
On the left-hand side, time performances pre-recalibration between 10 and 16 years old on 50m Freestyle male swimmers in 50m pool. Horizontal bars (gold) in each age represent the threshold for the 10 Best performances (pink points). On the right-hand side, time performances post-recalibration between 10 and 16 years old on 50m Freestyle male swimmers in 50m pool. Horizontal bars in each age still represent the threshold for the 10 Best performances pre-recalibration. Three types of performances constitute the new 10 Best performances: The ones in the 10 Best pre and post-recalibration (golden points), the ones in in the 10 Best only pre-recalibration (and present on the previous graph, pink points) and the ones in the 10 Best only post-recalibration (green points). The blue points are those above the threshold (10^th^ performance) after recalibration and the red points below the threshold represent the blue and green points before recalibration. The rest of the points (grey points) illustrate the other recalibrated points that are below the threshold.

Swimmers’ performances for all 10-years-old swimmers up to 17-years-old swimmers before applying the rebalancing method are represented on the left-hand side of **Fig 2**. The performances improve over time in a same-age category but as the absolute value of the balancing coefficient decreases, performances are less rebalanced year-by-year. On the right-hand side of **Fig 2**, rebalanced performance times are illustrated. We note that the 10 Best post rebalancing method is composed by new swimmers who are younger in their age categories. Indeed, the number of swimmers present in both 10 Best is low, as indicated by the stability rate (2, 2, 3 respectively for 10-years-old, 11-years-old and 12-years-old categories).

**Fig 3:**
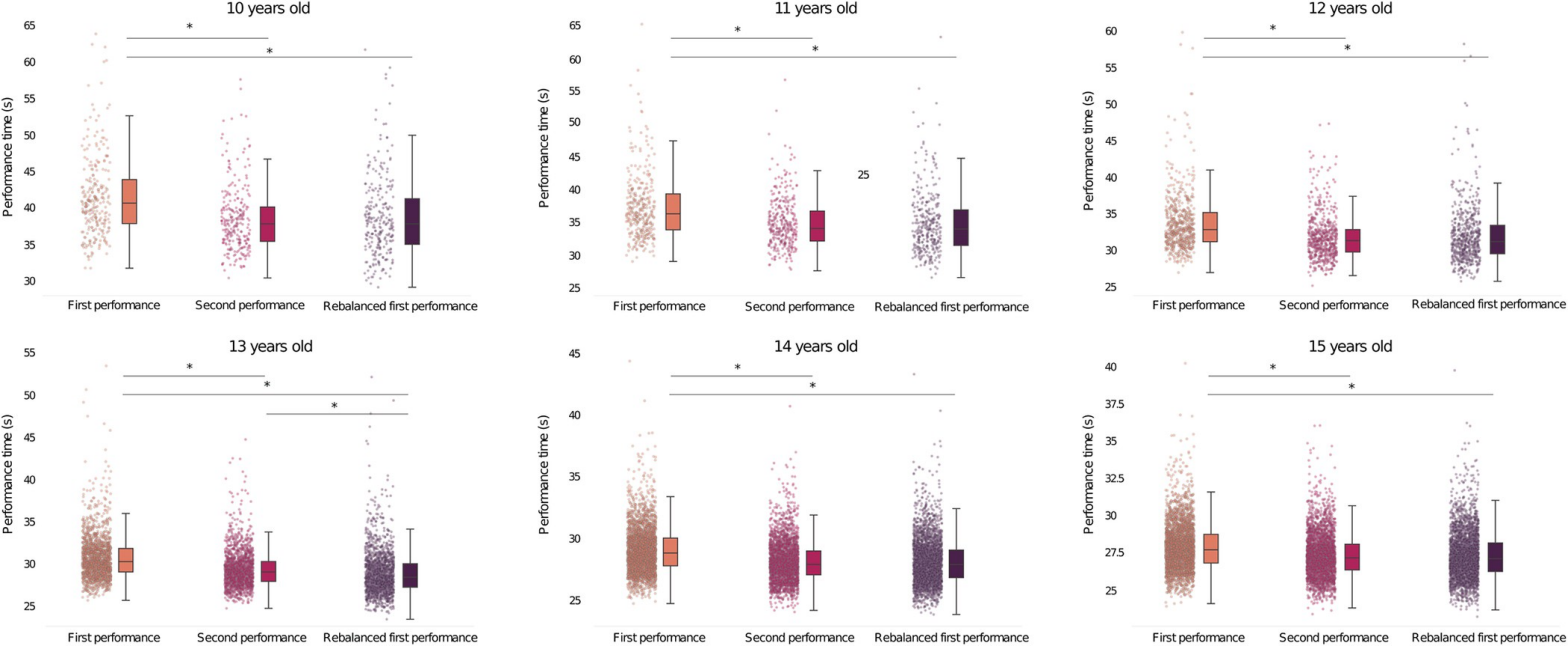
Statistical comparison of first, second and relocated performances. *: Significant mean performance difference (p < 0.01).

‘All’ swimmers’ first, rebalanced and second performance times are represented for 10 years old to 15 years old categories (**Fig 3**). All swimming times decreased with age generating a decrease in the confidence intervals. Once the Wilcoxon test was executed, we did not find any significant difference between rebalanced and second performance times for the 10 years old, 11 years old, 12 years old, 14 years old and 15 years old categories. Indeed, for these categories, the average of relocated first performances are respectively 39.47, 35.15 and 32.28 seconds and the average of second performances are 39.06, 35.05 and 32.06 seconds.

## Discussion

This study reveals RAE in French male and female swimmers in all types of swimming events from the 10 years old to the 17 years old categories. The RAE increases with the level of performance but tends to decrease in the older categories. A new corrective adjustment procedure has been implemented to rebalance the performance of young French swimmers. It has been tested on real performances, which allowed to validate this new method.

In our results, the proportion of swimmers born in the last semester (S2) is constantly lower than the proportion of the ones born during the first semester (S1). For example, at 12, 57.2% of male Freestyle swimmers were born during the first half of the year. This is in line with Cobley et al. [7] who studied Australian swimmers between 13 and 16 years, by level of competitiveness, but with a smaller sample. The largest difference between the first and second birth semester was found among the top 10%. Indeed, the proportion for the age of 13, 14, 15 and 16 were for the first semester 80%, 78.6%, 85% and 63.7% respectively. This unequal distribution is also present in Costa et al. [8] where they looked at the top 50 Portuguese male and female swimmers in each event born between 1992 and 1998 and aged between 12 and 18 years. The authors showed that those born in S1 represent 65.08% (13 years old), 67.80% (14 years old), 63.08% (15 years old), while 34.92% (13 years old), 32.20% (14 years old) and 36.92% (15 years old) of the population were born in S2. In this study, it can be seen that the proportion born in the last semester is almost equal to the proportion born in the first quarter.

Among female swimmers, in this same study, 43.11% of the 12 years old category was born in Q1 compared to 9.13% in Q4. In our analysis, in female swimmers, RAE is also present in all swimming events. Abbott et al. [17] identified RAE among Australian female breaststroke events (100m and 200m) between 12 and 14 years old with the inclusion criterion of having performed at least 5 times between 1999 and 2017 and having achieved a national level qualifying time in the events considered. Globally, as much as the level of competitiveness increases, RAE impact increases as well as the proportion of born in Q1. Indeed, for the 13 years age-group, in 200m Breaststroke, this proportion stands at 34.49% for ‘all’, 44.44% for top25% and 50.00% for top10%. In our findings, for a same age category, when observing the evolution of birth quarters repartition, it is worth noticing that the proportion of swimmers born in Q4 decreases as the competitiveness level increases. Our study exhibits greater effects with the level of competitiveness in accordance with previous studies [7].

Indeed, our technique is different from that used by Cobley et al. [7]: even if the progression rates are not linear [20,21], we have chosen to run linear regressions on each age category. Having a unique coefficient for each age category allows the coefficients to be independent of the previous and next age categories. In previous studies [17] authors applied a polynomial regression, but our aim was here to capture the importance of RAE within each category and not to have it affected by the performance of younger or older age categories.

Then, the novelty of this study lies mainly in the method, which does not only serve to rebalance the RAE but to adjust the performance of each swimmer according to the importance of the RAE within his/her category, and also to distance, stroke and sex. This allows all swimmers in the same category to be compared more objectively. Also, this is the first study to propose a rebalancing method on more than 5 million data using the exhaustivity of the competitions between 2000 and 2019, from the local competition level to the international one.

In our regressions, we observe a negative relationship that we can interpret as follows: in average, in a same-age category, the older the swimmer, the faster the performance time. This goes in the direction of the ‘maturation-selection’ hypothesis by Cobley [4] where the older ones have higher probabilities to be selected in representative teams because of the relative advantage associated to age differences. In the rebalancing procedures, swimmers who compose the new 10 Best are relatively young and are located at the beginning of their year of age. This seems logical because they benefit from a larger rebalancing coefficient. As the coefficient decreases, while age increases, we observe that the performances are less rebalanced and the 10 Best become more stable.

However, when relocating according to the competitiveness level, the performances dispersion is reduced, the population is smaller and so is the calibration coefficient. This is why the performances in the top 10% are poorly rebalanced. It is also possible that these performances occur when swimmers are relatively old in their age category, which is another reason for low rebalancing. This type of relocations has been previously shown in the study of De Larochelambert et al. [19], which confirmed the presence of a RAE in French alpine skiing with calibration coefficients decreasing with age.

This method rebalances performances in order to reveal the potential of each swimmer as opposed to previous studies which aimed at canceling RAE. Other variables should then be integrated, such as anthropometric variables, or power indices. In fact, swimming personal best times were found to be correlated with biometrics, such as height and weight [22], hand, forearm or torso lengths [23], or with muscle power assessed through standing broad jump or 30m running sprint time [24].

Williams et al. [25] have highlighted four groups of potential predictors of adult success in football: physical, psychological, skill and sociological variables. In addition, they specially focus on maturation, life events (as birth place or information about siblings), developmental environment and external (non-sporting) environment. Indeed, performance prediction is a complex process. Potential identification should progressively integrate most of these co-factors.

## Limits

Our method is based on the slope coefficient of the linear regression between performance average and additional months. We acknowledge that we cannot be certain of the underestimation or overestimation of the coefficient. The greater the dispersion, the more the best swimmers will be overestimated while the less performant swimmers will be underestimated. We could obtain slope coefficients by stratifying by competitiveness levels, but, within groups, there will still be dispersion. Some will, once again, be overestimated compared to others and vice versa. Ideally, an individual coefficient should be estimated, but in practice it might be difficult to obtain, as it would take larger dataset for a unique swimmer to establish his/her own performance-relative age gap relationship.

## Conclusion

We demonstrate the presence of relative age effect in both male and female French young swimmers. This highlights the importance of a relative age effect, which increases with competitiveness level. A corrective adjustment procedure allows to more objectively monitor the performance realized by each swimmer with respect to chronological age. This method may allow for a better understanding of each swimmer performance and improve the selection process in the early quest for high potentials. If used on the field, this method can help overcome differences in the chronological age of swimmers and can be used as a decision support tool for coaches by allowing them to visualize possible differences among swimmers within the same age category.

## Supporting information

S1 File Contains all the supporting tables and figures. (DOCX)
