# Supplementary material for: Corrective adjustment methods for relative age effects on French young swimmers’ performances
Source: PLoS One. 2023 Apr 24;18(4):e0283229. doi: 10.1371/journal.pone.0283229 (PMC10124878; doi:10.1371/journal.pone.0283229)
Supplement: S1 File — (DOCX) [file pone.0283229.s001.docx]

**Supporting information:**

***S1 Table****: Birth quartiles distribution in male individual events in 25 and 50m pool for all age categories between 10 and 18 years old (Q1-4: birth quarters, χ²: Chi-Square value, ***: P < 0.01, OR: odds ratios between Q1 and Q4, 95% CI: 95% Confidence Interval.)*

***S2 Table:*** *Birth quarters distribution in female individual events in 25 and 50m pool (Q1-4: birth quarters, χ²: Chi-Square value, ***: P < 0.01, OR: odds ratios between Q1 and Q4, 95% CI: 95% Confidence Interval.)*
